# Numerical investigation of co-flow jet integration to enhance the aerodynamic efficiency of airfoils used in wind turbine applications

**DOI:** 10.1038/s41598-026-38769-0

**Published:** 2026-03-12

**Authors:** Mohamed B. Farghaly, Ossama M. Abd El Kader, Abdullah M.A. Alsharif, Mohamed Halawa

**Affiliations:** 1 Mechanical Engineering Department, Faculty of Engineering, Fayoum University, El- Fayoum, 63514 Egypt; 2 Department of Mechanical Engineering, College of Engineering in Yanbu, Taibah University, Yanbu Al-Bahr, Saudi Arabia; 3 Mechanical Engineering Department, Faculty of Engineering, Al-Azhar University, Cairo, Egypt

**Keywords:** Co-flow jet, Wind turbine airfoil, S809 airfoil, CFD, Suction slot location, Injection slot angle, Energy science and technology, Engineering

## Abstract

Co-Flowing Jet (CFJ) is considered one of the most significant active flow controls techniques used to migrate airfoil vortices and delay stall phenomenon. This research presents a computational investigation of the S809 airfoil equipped with CFJ to predict the optimum configuration for improved aerodynamic performance. The effect of injection angles, suction location, and injected mass flow rates (m^o^) were analyzed. Several suction slots were studied at different locations of (60, 70, 80 and 90) %C for various injection angles of 48°, 58°, 68°, 78° and 88°. The most effective combinations of suction location and injection angle that achieves the maximum (C_l_/C_d_) ratio were determined and then analyzed for different injected (m^o^) of 2.5%, 5% and 7.5%. The results indicate that the highest lift-to-drag ratio (C_l_/C_d_) is achieved with a suction slot at 80%C and an injection angle of 78°. Increasing the injected mass flow rate beyond 2.5% showed negligible improvement in airfoil performance. Furthermore, a CFJ turn-off condition was evaluated to assess the jet channel effect, revealing a 42% reduction in lift, increased drag, and earlier stall occurrence at an attack angle of 16.24°. Overall, the findings confirm that CFJ significantly enhances the aerodynamic characteristics of wind turbine airfoils under optimal configurations.

## Introduction

Traditional energy sources, including oil, natural gas, and coal, have historically played a pivotal role in supporting economic growth. Nevertheless, the accelerated depletion of these resources, coupled with the rising global demand for energy, led to an increase of approximately 1.8% in primary energy consumption at the beginning of 2012^1^. Due to growing environmental concerns, many organizations have promoted intensive research into the development of more efficient and environmentally friendly power plants that incorporate advanced technologies. With increasing emphasis on environmental protection, renewable energy sources and clean fuel technologies such as wind power are receiving significant attention as viable alternatives to conventional energy systems^2^. Wind power is a clean, inexhaustible, and renewable energy source. It has served humanity for many generations, historically being utilized to propel ships, operate windmills for grinding crops, and pump water, and in modern times to drive wind turbines for electricity generation^3^. Recently, increasing attention has been directed toward the utilization of wind energy as an alternative source of power to reduce reliance on fossil fuels, as it is considered one of the most cost-effective and widely available renewable resources^4^. Wind energy holds strategic significance in the field of power generation, as it contributes to restructuring the energy mix, reducing environmental pollution, and promoting sustainable development within the energy sector^5^.

Wind turbines are devices designed to extract kinetic energy from the wind, and their performance has been extensively investigated in literature. The most critical aerodynamic parameter influencing wind turbine efficiency is the output power. At very high wind speeds, power output must be limited to prevent structural failure of the rotor blades and protect the electrical generator system. Conversely, at low wind speeds, structural limitations are less critical, and maximizing output power becomes essential. Typically, wind turbines are designed to achieve optimal performance at a specific tip-speed ratio relative to the free-stream wind velocity. Velocity when the free-stream velocity deviates from this design point, efficiency decreases, which can be compensated by adjusting the rotational speed or the blade pitch angle. At low wind speeds, enhancing power output requires improving the aerodynamic lift coefficient and the lift-to-drag ratio, often achieved by increasing the angle of attack. Nonetheless, the angle of attack is constrained to avoid blade stall or to delay flow separation^6^.

Farghaly et al.^7^ investigated the performance characteristics of diffuser-augmented wind turbines (DAWTs) were analyzed computationally using the ANSYS Workbench package. The system consists of a simple shrouded diffuser with a flange at the exit periphery and a wind turbine rotor positioned inside it. The influence of this configuration on enhancing turbine performance characteristics, including output power generation and the power coefficient, was systematically investigated. Farghaly and Aboelezz^8^ analyzed the impact of the dynamic stall phenomenon on Horizontal Axis Wind Turbines (HAWTs) under yaw misalignment conditions through mathematical analysis. A model was developed using the MATLAB commercial package, based on the Blade-Element Momentum Theory (BEMT). Computational results obtained with the Beddoes–Leishman model in a yawed configuration were applied to the HAWT and integrated into the developed code for yawed flow analysis. The study predicted the influence of dynamic stall on both the normal (C_n_) and tangential (C_t_) force coefficients. They reported that yawed flow conditions increased tangential force fluctuations by up to 15% and altered the normal force coefficient by 12% compared to aligned flow conditions. Abdelghany et al.^9^ studied the influence of wind speed on the behavior of dynamic stalls and the performance characteristics of HAWTs under yaw misalignment using BEMT in combined with the Beddoes–Leishman model in a yawed configuration. The S809 airfoil was employed for designing the blade profile. Performance variations with respect to flow angles and rotor azimuthal angles were examined at different wind speeds across various spanwise sections of the rotor blade. The results obtained observed variations in lift coefficient of up to 18% across different spanwise sections. Finally, appropriate flow control techniques were recommended for the identified critical regions.

Conventional airfoil sections may not be sufficiently suitable to meet the operating requirements of wind turbines in diverse environmental conditions. Therefore, flow control for wind turbine airfoils has become a critical area of research, aiming to reduce drag, enhance lift, minimize noise, prevent flow separation at high angles of attack, and delay stall^5^. Flow control systems are generally categorized into two main types: passive and active. These systems are employed to achieve desirable modifications in the boundary layer and flow patterns, with the objectives of increasing lift, reducing drag, and suppressing flow separation^10^. In passive flow control (PFC) systems, energy is transferred from the main airflow to the boundary layer. In contrast, active flow control (AFC) systems require an external energy source. In AFC, energy is supplied from external resources such as compressors or plasma discharge devices into the working fluid. Passive methods are generally easier to implement and less costly compared to active methods; however, their influence on overall efficiency is relatively limited. Active flow control techniques, on the other hand, provide more substantial improvements in aerodynamic performance parameters but are less commonly applied due to their greater complexity. Nevertheless, recent studies have demonstrated their significant potential and enhanced impact on overall effectiveness and power output.

Among passive techniques, vortex generators (VGs) are widely used due to their proven efficiency and ease of deployment. Barrett et al.^11^ conducted experimental investigations on a smart vortex generator that is deployed near the stall angle of attack (AoA) and remains retracted under other wing operating conditions. The results demonstrated that lift fluctuations could be reduced by approximately 10–12% while delaying stall by 2–3 degrees. Slats and flaps are commonly employed in both aviation and wind turbines to enhance lift. In aircraft, they are primarily used to reduce take-off and landing distances and times. The deployment of flaps and slats increases the Camber and, in many cases, the wing planform area, thereby improving lift generation. The plain flap was the first type introduced in aircraft; despite its relatively low aerodynamic efficiency, it remains in use today due to its simplicity and ease of implementation. Trailing-edge flaps with various deflections have also been applied as a passive flow control technique to improve aerodynamic performance and modify stall behavior on blades, particularly near the blade tip. This approach has been examined by Farghaly et al.^12^, through computational analyses across a range of wind speeds for horizontal-axis wind turbines (HAWTs). The results showing that deflection of 15° increased lift coefficient by 6–8% and improved the lift-to-drag ratio by 5%. Abdelghany et al.^13^ analyzed the effect of winglet height on the aerodynamic characteristics of Horizontal-Axis Wind Turbine (HAWT) blades through computational analysis. A turbine blade with an optimized profile was employed to construct rotor geometry. Different winglet heights relative to the blade radius (WHLR) were evaluated at a cant angle of approximately 90 degrees and a constant tip-speed ratio. The results demonstrated that the incorporation of tip winglets led to improved rotor performance and optimized winglets increased power output by 4–7% and reduced tip vortex losses.

To improve aerodynamic effectiveness and efficiency, slotted flaps were developed with a gap between the flap and the main wing. Harris et al.^14^ investigated various NACA airfoil sections equipped with a single slotted flap. Modern wing designs frequently incorporate various combinations of slats and flaps. An experimental study by Morgan Jr^15^. demonstrated that the combined use of slats and flaps shifts the lift coefficient (C_l_) versus angle of attack (AoA) curve upward by approximately 1.5.

Several PFC methods have been explored to enhance aerodynamic performance. One such method, inspired by natural forms, is the tubercle passive flow control technique, which mimics the morphology of whale flippers. The influence of tubercles on the aerodynamic behavior of wind turbines has been investigated in multiple studies. Incorporating a sinusoidal tubercle along the leading edge has been shown to mitigate stall severity and reduce periodic loading. Results indicate that turbine blades with wavy leading-edge profiles exhibit improved power output with increasing wavelength. Tubercle-inspired leading edges were investigated by Huang et al.^16^ and Bai et al.^17^, revealing that sinusoidal leading edges improved stall margin by 5°–6° and increased power coefficient by 3–5% at moderate wind speeds. However, at low wind speeds, the tubercle leading edge may reduce power generation due to premature boundary-layer separation^16–19^. AFC is a relatively recent development that has demonstrated the ability to significantly extend the operational range of wind turbines by delaying flow stalls. In addition, it offers substantial potential for increasing power output and overall efficiency. Unlike passive techniques, AFC requires an external energy source. A key advantage of AFC its adaptability, as it can respond to variations in inflow conditions and can be deactivated when its effects are no longer needed^18^. In recent years, AFC has attracted significant attention as an effective approach for enhancing the aerodynamic characteristics of wind turbines^20^, including synthetic, flow control airfoil, plasma flow control^21^ and newly Co-Flow Jet (CFJ)^22– 23^.

Wang et al.^24^ employed unsteady blowing jets on a wind turbine airfoil and demonstrated that the variation in lift was reduced both in the absence and presence of large-scale unsteadiness in the free stream flow and the results observed a 7% reduction in lift fluctuations in the presence of free-stream unsteadiness. Kang and Park^25^ investigated steady blowing jets on the S809 airfoil, resulting in an increase in the maximum lift coefficient and a delay in stall onset and reported an increase in maximum lift coefficient by 9% and a delay in stall onset by 3°–4°. However, these analyses of blowing jets as an AFC technique did not address how the required mass airflow rate for blowing systems in wind turbines could be supplied. Maldonado et al.^26^ employed a zero-net-mass-flux synthetic jet on the S809 airfoil under dynamic stall conditions. The results demonstrated that flow reattachment was improved during both pitch-up and pitch-down motions at an angle of attack (AoA) of 15°, with lift increases of 6–8%.

Zha et al.^27–30^ introduced a modern flow control technique by applying a CFJ on the low-pressure surface of the airfoil, demonstrating a remarkable ability to significantly enhance stall margin, increase lift, and reduce drag. The CFJ mechanism energizes the boundary layer by extracting air from the low-pressure region near the trailing edge and re-injecting it into the high-pressure region near the leading edge through a small, embedded compressor within the airfoil structure. The results indicated that the boundary layer remained stable over a longer duration and across a wider range of angles of attack, thereby delaying stalling and enabling the turbine to operate efficiently at lower wind speeds. The results showing lift enhancement of 8–12%, drag reduction of 5–7%, and stall delay up to 5°, achieved by energizing the boundary layer via suction and injection slots connected to an embedded compressor. The CFJ method consists of two slots: an injection slot located near the leading edge on the upper surface of the airfoil, and a suction slot positioned near the trailing edge, also on the upper surface. To maintain zero net mass flux, the mass flow rate of the injection and suction streams must be equal. G. C. Vishnukumar and C. M. Vigneswaran^31^ examined the significant influence of the locations and dimensions of these suction and injection slots on aerodynamic performance. Akter et al.^32^ developed a modified airfoil derived from the NACA 23,012 and analyzed it using a high-fidelity panel method at Re = 2 × 10⁶. Several geometric parameters were varied and evaluated in XFLR5, and the numerical model was validated against Miley’s experiments with errors below 4%. The optimized design achieved a 7% increase in lift, a 5.2% improvement in lift-to-drag ratio, and a 46% reduction in moment coefficient, with further 3D analysis confirming its suitability for light-aircraft applications.

In general, most of the above-mentioned, AFC methods are designed to enhance performance near or slightly beyond the stall point, thereby extending and enlarging the operational margin while simultaneously increasing lift, particularly in the high angle-of-attack regime. Delaying stall phenomena contributes to increased lift, reduced drag, and improved overall aerodynamic performance of wind turbine airfoils, ultimately enhancing the efficiency of wind turbine blades. Despite the extensive studies that have been conducted on active flow control (AFC) techniques for wind turbine airfoils, a clear understanding of the aerodynamic behavior of the S809 airfoil under Co-Flow Jet (CFJ) application has not yet been fully established. The combined effects of suction-slot location and injection-slot angle have not been systematically quantified in previous work, and the influence of CFJ system failure on the baseline aerodynamic performance has also been insufficiently addressed. Furthermore, the impact of varying injected mass-flow rates within a CFJ cycle has not been comprehensively examined for wind-energy applications.

To address these gaps, emphasis is placed in this study on developing a detailed aerodynamic assessment of the S809 airfoil equipped with CFJ flow control, with particular attention directed toward identifying the most influential geometric parameters governing CFJ effectiveness. In addition, a dedicated evaluation of CFJ turn-off behavior is included to ensure that the implications on operational stability and safety are explicitly clarified.

To perform this investigation, a computational simulation domain was formed by ANSYS-Fluent package, and the models of turbulence were validated for several models compared with baseline airfoil experimental measurements data. The computational simulation model was resolved for airfoils shape with and without Co-Flow Jet slots to produce optimum CFJ geometrical shape of airfoil. Various suction slots were studied at different locations, also, the injection slot was studied at various angles. The effect of injection slot angle and suction slot location have been studied and analyzed. The most effective suction slot’s location and injection slot angle were determined and then analyzed for different injected mass flow rates and then, the most effective mass flow rate to facilitating compressor was determined. Finally, to predict the jet shape effect on the aerodynamic characteristics of baseline, a CFJ turn-off condition was analyzed, and the obtained results were discussed.The novelty of this work is established through the first integrated passive flow control investigation that correlates suction-slot location, injection-slot angle, and CFJ operating conditions for the S809 airfoil. Through this contribution, new insight is provided into optimizing lift enhancement, drag reduction, and stall-delay mechanisms for wind-turbine aerodynamic applications.

## Numerical modelling

Computational Fluid Dynamics (CFD) is a powerful tool for studying and analyzing fluid dynamics problems without the need for physical prototypes. Rather than constructing and testing experimental models, CFD utilizes computer-aided simulations to predict fluid behavior with high accuracy. This technique relies on fundamental computational methods, including mathematical modeling and numerical analysis. Owing to its cost-effectiveness and reliable accuracy, CFD has become widely applied across diverse fields of energy and fluid sciences. In this study, the ANSYS Fluent package is employed as the primary CFD tool to validate the S809 airfoil model against experimental data, ensuring that the numerical predictions are consistent with physical results. Following validation, the model is used to investigate various parameters of CFJ configurations to determine the optimal blade shape that maximizes lift, enhances power output, and delays stall.

### Governing equations

The investigation of various turbine airfoil shape designs was carried out using both steady and unsteady two-dimensional Reynolds-Averaged Navier–Stokes (RANS) equations, implemented through the commercial software ANSYS Fluent. A coupled scheme was adopted for velocity–pressure coupling, while a second-order discretization approach was employed to enhance solution accuracy. To determine the most reliable turbulence model, three alternatives were evaluated: the Realizable k–ε model, the Spalart–Allmaras model, and the k–ω (SST) model. Once the CFD model was developed and the solution process was completed, it became possible to systematically analyze a wide range of parameters by repeatedly solving the numerical problem. The continuity can be written as illustrated in Eq. (1), and momentum equations in y, x directions can be written as illustrated in Eqs. (2, 3)^33^.

1 $$\frac{{\partial \left( {\rho \vec{u}} \right)}}{{\partial x}} + \frac{{\partial (\rho \vec{v})}}{{\partial y}} = 0$$

2 $$\:\frac{\partial\:\rho\:\overrightarrow{u}\overrightarrow{u}}{\partial\:x}+\frac{\partial\:\rho\:\overrightarrow{u}\overrightarrow{v}}{\partial\:y}=-\frac{\partial\:p}{\partial\:x}+\frac{\partial\:}{\partial\:x}\left(\lambda\:\nabla\:.\overrightarrow{V}\:\:+\:2\mu\:\frac{\partial\:u}{\partial\:x}\:\right)+\frac{\partial\:}{\partial\:y}\left(\mu\:\left(\frac{\partial\:v}{\partial\:x}+\frac{{\partial\:}^{\underset{u}{\to\:}}}{\partial\:y}\right)+\rho\:{B}_{x}\:\right)$$

3 $$\:\frac{\partial\:\rho\:\overrightarrow{u}\overrightarrow{v}}{\partial\:x}+\frac{\partial\:\rho\:\overrightarrow{v}\overrightarrow{v}}{\partial\:y}=-\frac{\partial\:p}{\partial\:x}+\frac{\partial\:}{\partial\:x}\left(\lambda\:\nabla\:.\overrightarrow{V}\:+\:2\mu\:\frac{\partial\:v}{\partial\:x}\right)+\frac{\partial\:}{\partial\:y}\left(\mu\:\left(\frac{\partial\:\overrightarrow{v}}{\partial\:x}+\frac{\partial\:\overrightarrow{u}}{\partial\:y}\right)+\rho\:{B}_{y}\:\right)$$

Where;

4 $$\:\overrightarrow{V}=\overrightarrow{u}\:\:\:{n}_{x}\:+\:\overrightarrow{v}\:\:{n}_{y}$$

The integration formulas of Reynolds-averaged-Navier-stokes (RANS) equations containing the momentum and continuity that created as shown below^34^:

5 $$\:{\oint\:}_{\partial\:\varOmega\:}\rho\:Vds=0\:$$

6 $$\:\frac{\partial\:}{\partial\:t}{\oint\:}_{\varOmega\:}\overrightarrow{w}\:d\varOmega\:+{\oint\:}_{\partial\:\varOmega\:}\overrightarrow{J}\:ds\:=0$$

Where ρ represents the density, Ω is the surrounded volume by the control surface ∂Ω for 2d study control surface, V represents the perpendicular velocity to element of surface ds,$\:\:\overrightarrow{W}$ is the vector of conservative variables, and $\:\:\overrightarrow{J}$ involves of the diffusive and convective fluxes which created as shown below^35^:

7 $$\:\:\overrightarrow{W}=\left[\begin{array}{c}\rho\:u\\\:\rho\:v\end{array}\right],\:\overrightarrow{J}=\left[\:\:\:\begin{array}{c}\rho\:uV+{n}_{x}p-\left(\mu\:+{\mu\:}_{t}\right)\left({n}_{x}\frac{\partial\:u}{\partial\:x}\:+{n}_{y}\frac{\partial\:v}{\partial\:y}\right)\\\:\rho\:uV+{n}_{y}p-\left(\mu\:+{\mu\:}_{t}\right)\left({n}_{x}\frac{\partial\:v}{\partial\:x}\:+{n}_{y}\frac{\partial\:v}{\partial\:y}\right)\end{array}\:\:\:\right]$$

Where $\:{\mu\:}_{t}$ and $\:\mu\:$ represents the turbulent and laminar viscosity respectively. The equations of flow (4 and 7) are resolved by pressure-based scheme offered by Lestari and Rajagopalan^36^ to simulate the viscosity of turbulent ($\:{\mu\:}_{t}$). Also, the variables of flow are normalized as described in^37^. By numerically resolving the equations mentioned above, the needed parameters of flow such as velocity components and pressure can be calculated, and hence, the streamlines, coefficients of lift and drag can be found. Finally, the enhancement in the aerodynamic coefficient such as C_l_, C_d_ and C_l_/C_d_ due to using the co-jet flow techniques can measure by the following relations.

8 $$\eta \left( {{C_l}} \right)={\text{ }}\left( {{C_l}{,_{CFJ}}--{\text{ }}{C_l}{,_{Baseline}}} \right)/{\text{ }}{C_l}{,_{Baseline}}$$

9 $$\eta ({C_d}){\text{ }}=\left( {{C_d}{,_{CFJ}}--{\text{ }}{C_d}{,_{Baseline}}} \right)/{\text{ }}{C_{d,}}_{{Baseline}}$$

10 $$\eta ({C_l}/{C_d}){\text{ }}=\left( {{C_l}/{C_{d,{\text{ }}CFJ}}--{\text{ }}{C_l}/{C_d}{,_{Baseline}}} \right)/{\text{ }}{C_l}/{C_d}{,_{Baseline}}$$

### Model description and computational domain

**Fig. 1:**
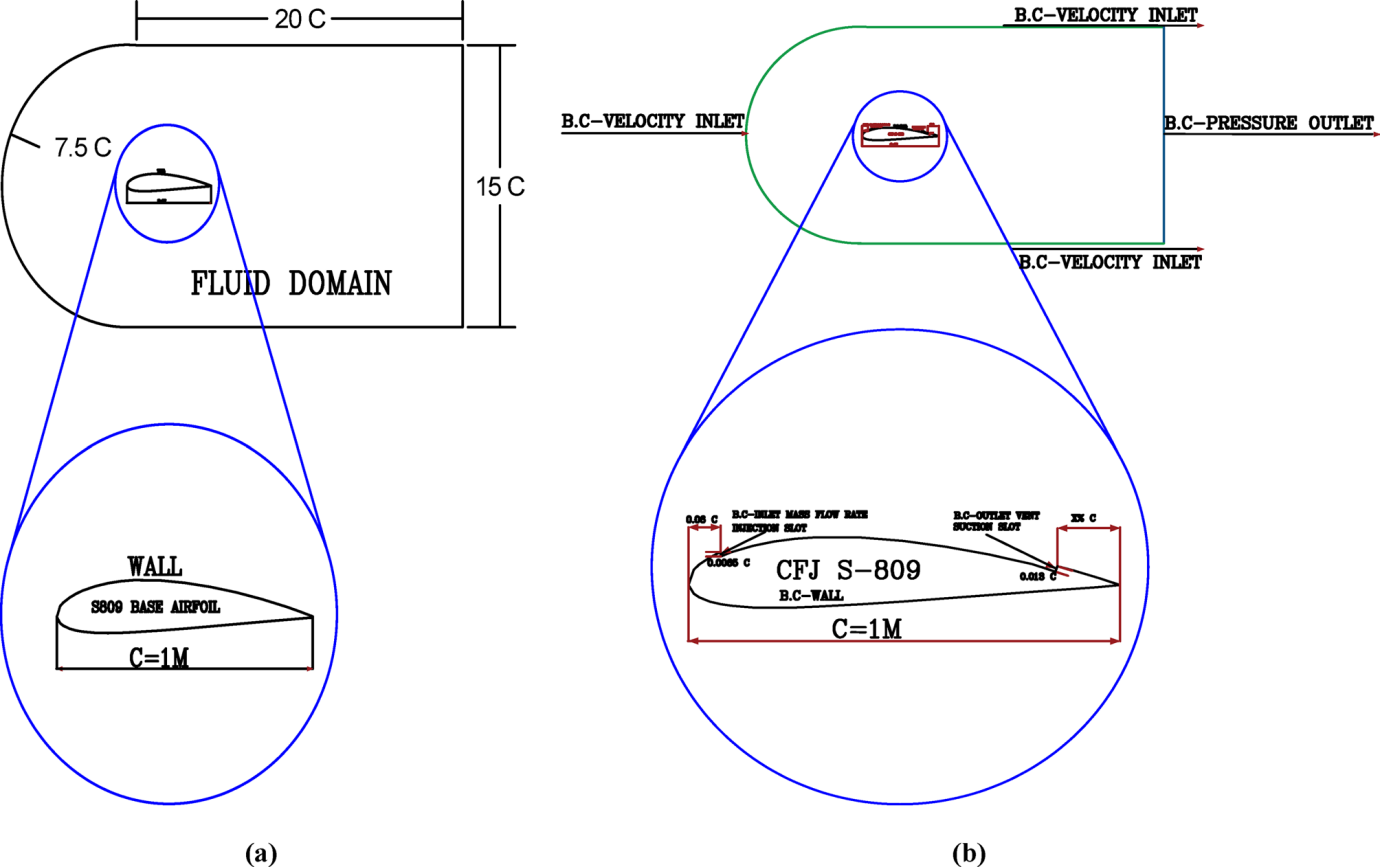
Details dimensions of simulation model, (**a**) Computational domain dimensions and Base S-809 airfoil boundary conditions, (**b**) Fluid domain and Co-Flow-Jet model boundary conditions.

The CFJ technique was implemented on the baseline S809 airfoil, which is widely used in the wind turbine industry. The mechanism consists of injection slots positioned near the leading edge and suction slots located near the trailing edge, both placed on the suction surface of the airfoil. According to G. C. Vishnukumar and C. M. Vigneswaran^31^, the configuration is most effective when the suction slot length is approximately twice that of the injection slot. In this study, the injection slot was placed at about 6% of the chord length, utilizing the local geometric region near the leading edge. The injection angle was treated as a geometrical parameter (P_1_), with several values analyzed to assess its influence. To determine the optimal configuration, the suction slot location was also treated as a geometrical parameter (P_2_) and investigated at multiple positions, while the suction slot angle was fixed perpendicular to the tangential line at the suction surface. The detailed dimensions of the computational domain, the baseline S809 airfoil, and the CFJ models are illustrated in Fig. 1(a, b), and the model description is summarized in Table 1.

## Solution setup

ANSYS Fluent software was employed to solve and simulate the fluid domain for both the baseline S809 airfoil and the enhanced S809 configuration with CFJ techniques. A coupled scheme was selected for velocity–pressure coupling, in combination with a second-order upwind discretization to improve solution accuracy. The choice of turbulence model was a critical step, as the k–ω SST, Realizable k–ε, and Spalart–Allmaras models are widely applied in similar aerodynamic studies. Therefore, all three models were assessed to identify the one that best captured the relevant flow characteristics, after which the selected model was applied to analyze the remaining study parameters. To validate the numerical approach, both turbine configurations were simulated, and the results were compared against available experimental data^38^. The complete ANSYS Fluent setup is summarized in Table 2.

### Boundary conditions

The definition of boundary conditions is a critical step in CFD simulations, as it has a significant impact on the accuracy of the obtained results; therefore, the conditions were carefully selected. Using the ANSYS Fluent package, the pressure, flow field, velocity, and temperature around the S809 turbine airfoil were analyzed within a C-shaped computational domain, as illustrated in Fig. 1. The free-stream air temperature was set to 288.16 K, corresponding to ambient conditions. At this temperature, the air density was taken as 1.225 kg/m³, the dynamic viscosity as µ = 1.7894 × 10⁻⁵ kg/m·s, and the pressure as 101,325 Pa, as summarized in Table 3. A uniform inlet boundary condition was assumed, with the free-stream wind velocity set to 14.6 m/s. At the domain outlet, a pressure-outlet boundary condition was applied, while the airfoil surface was modeled as a stationary wall. For the Co-Flow Jet configuration, the suction slot was defined as a mass flow rate inlet and treated as the study parameter P_3_ to determine the most efficient operating value. The relationship between the total incoming air mass flow rate through the wind turbine and the mass flow rate injected from the high-pressure slot (injection slot) was established. The inlet mass flow rate was expressed as a percentage of the total incoming mass flow, while both the total and injected mass flow rates were calculated using Eqs. (11) and (12), respectively.

11 $${M^o}=\raise.5ex\hbox{$\scriptstyle 1$}\kern-.1em/ \kern-.15em\lower.25ex\hbox{$\scriptstyle 4$} xpx{\text{ }}{D^2}x{\text{ }}V{\text{ }}xr$$

12 $${{\mathrm{m}}^o}={{\mathrm{P}}_3}{\mathrm{X}}{{\mathrm{M}}^o}$$

Where M^o^ represents the total incoming rate of mass flow reaching wind turbine, D is the wind turbine diameter (blade length multiplied by 2), V is the incoming air velocity, ρ represents the air density which equal 1.225 kg/m^3^, m^o^ is the injected mass flow rate from injection slot.

**Table 1:** Model description and computational domain.

Name	Description
Airfoil	S-809
Chord length	Unit
Computational domain shape	C shape with radius of 7.5 C
Computational domain dimension	20 C downstream and 15 C height
Injection slot location	6% C
Injection slot depth	0.0065 C
Suction slot depth	0.013 C
Injection angle	Parameter 1 (P_1_)
Suction slot location	Parameter 2 (P_2_)
Ratio of sucked or injected mass flow rate	Parameter 3 (P_3_)
Suction slot depth	1.3% C
Suction slot angle	Vertical on tangent to the airfoil surface

**Table 2:** Ansys fluent setup.

Name	Description
Fluid type	Air
Solver	Density based solver
Reynolds number	1 × 10^6^
Free-stream wind velocity	14.6 m/s
Turbulence model	K-W SST
Governing equation	01- Steady state with steady Reynolds averaged Navier-Stokes (RANS) are used for low angle of attack (From AoA − 10.1:18.1). 02- Unsteady Reynolds averaged Navier-Stokes (URANS) are used for high angle of attack (From AoA 20 and more)
Angle of attack	-10: 24 deg

**Table 3:** Computational domain boundary condition.

Boundary name	Boundary condition
Up stream	Inlet velocity of 14.6 m/s
Down stream	Pressure outlet
Airfoil surface	Stationary wall (no slip)
Suction slot	Outlet vent
Injection slot	Inlet mass flow rate 2.5%, 5% & 7.5% of incoming air flow

### Mesh independence and grid check

**Fig. 2:**
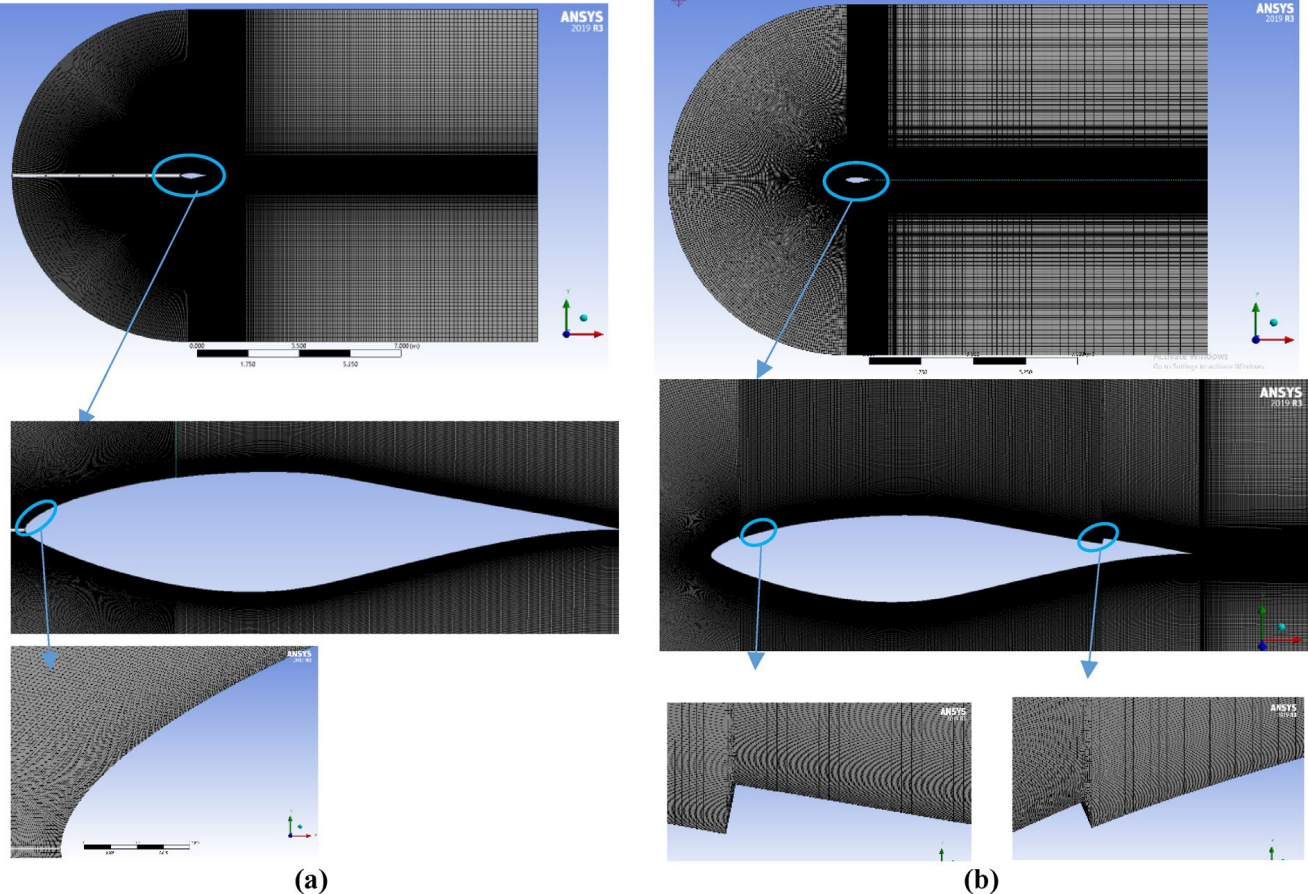
Meshing grid for Computational domain with different airfoil models, (**a**) Base S-809, (**b**) Co-Flow-Jet model.

To accurately resolve the aerodynamic behavior of a two-dimensional airfoil section used in wind turbine blades, a highly refined mesh was generated in the vicinity of the airfoil surface, with a gradual coarsening of grid density toward the far-field boundaries. The computational domain was discretized into finite elements to enable precise evaluation of local flow variables and to improve prediction of aerodynamic characteristics. In two-dimensional simulations, structured quadrilateral elements are typically preferred for their numerical stability, while unstructured triangular elements are commonly adopted to accommodate complex geometries. Previous studies by Sørensen and Farghaly highlighted the importance of optimized meshing strategies for achieving reliable numerical accuracy in airfoil simulations^12– 13,39–41^. In the present work, a C-type computational domain was generated using the ANSYS Meshing tool. Boundary-layer inflation layers were applied along the airfoil surface to refine the near-wall grid, enhance resolution in critical regions, and maintain a dimensionless wall distance of $\:{y}^{+}\le\:1$, enabling accurate capture of the boundary-layer development, as illustrated in Fig. 2. The mesh specifications are summarized in Table 4.

**Table 4:** Mesh specifications summary for base S-809 and CFJ airfoils.

Airfoil Type	BASE S-809	CFJ
Mesh type	Quadrilateral structured mesh	
Number of Elements (N.O.E)	500,054	540,474
Number of Nodes (N.O.N)	502,212	542,031
Minimum. orthogonal quality	0.641	0.8117
Minimum aspect ratio	1	1
First layer thickness	0.00025	0.000075
Near-wall resolution (y+)	≤ 1	≤ 1
Max skewness	0.556	0.7

**Fig. 3:**
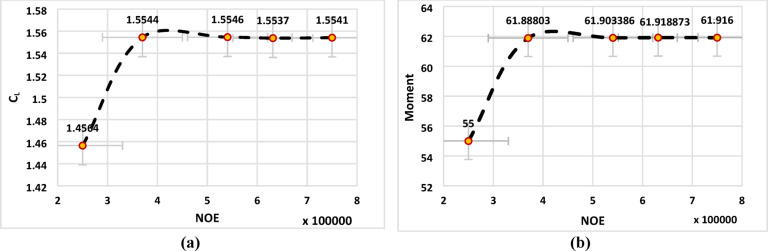
Mesh independent check, (**a**) Coefficient of lift, (**b**) Moment.

Increasing the number of computational cells generally enhances the accuracy and robustness of CFD predictions. However, excessive mesh refinement leads to a substantial increase in computational cost and may necessitate high-performance computing resources without providing proportional gains in solution accuracy. Therefore, an optimal balance between accuracy and computational efficiency must be achieved by identifying a mesh that yields grid-independent solutions. In this study, a systematic grid independence test was performed to determine the appropriate mesh resolution. Five different mesh densities were generated and evaluated by tracking the convergence behavior of two primary aerodynamic parameters: the lift coefficient and the pitching moment coefficient. Once both parameters exhibited negligible variation with further mesh refinement, the corresponding mesh was selected as the optimal grid and employed for all subsequent simulations. Figure 3 presents the effect of mesh density on the predicted lift coefficient and pitching moment coefficient for the S809 airfoil at an inlet velocity of 14.6 m/s and an angle of attack of 10.2°. Based on the grid sensitivity analysis, the mesh consisting of 540,474 elements was selected, as it provided grid-independent results while maintaining a reasonable computational cost.

### Validation of numerical results against published literature^38^

**Fig. 4:**
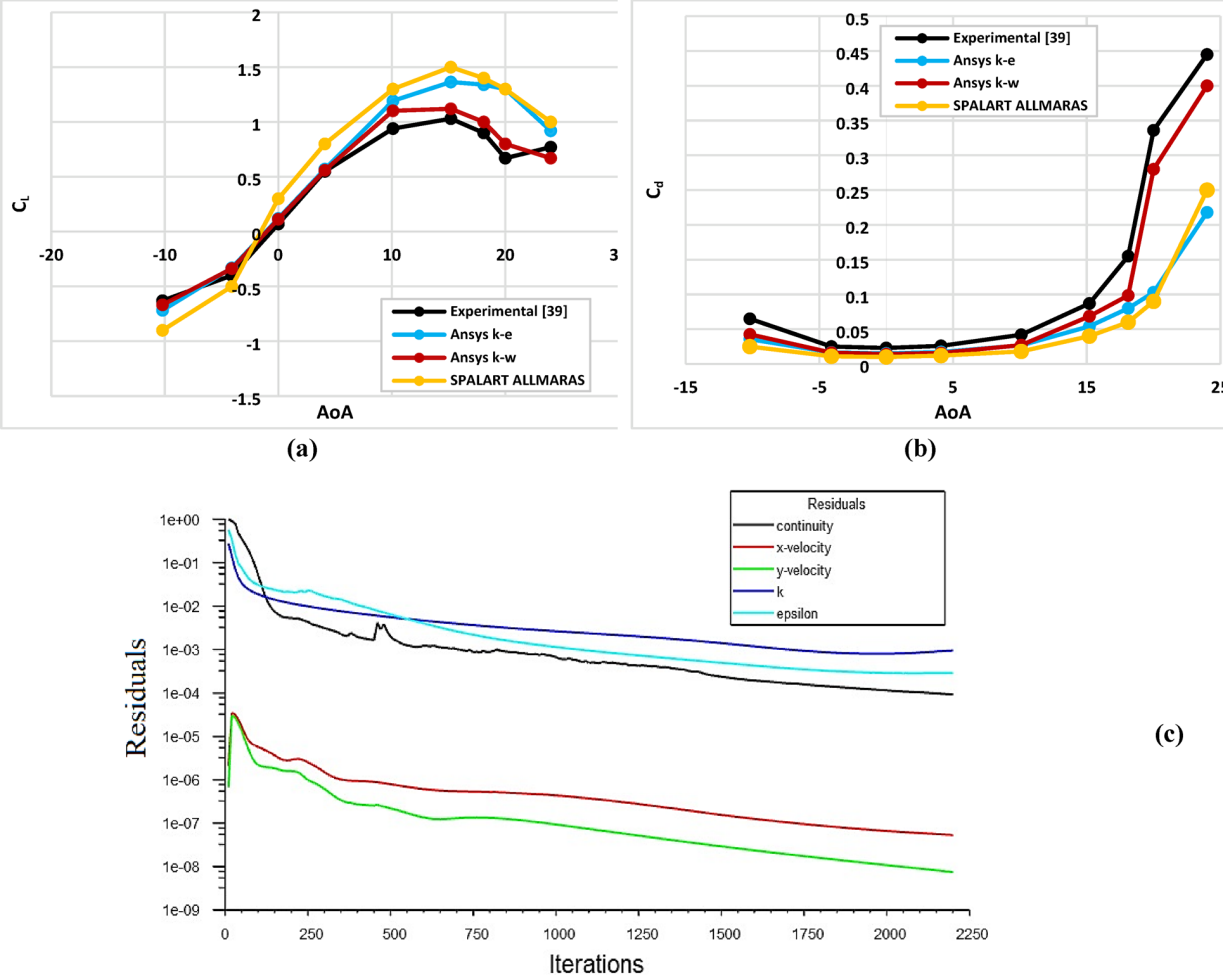
Computational domain validation, (**a**) Lift Coefficient, (**b**) Drag Coefficient^38^, (**c**) Convergence history.

To validate the reliability of the numerical framework for the baseline S809 airfoil and the CFJ-modified configuration, simulations were performed using three turbulence models: the Realizable k–ε, Spalart–Allmaras, and k–ω SST models. The numerical predictions were systematically compared with published experimental data obtained under comparable flow conditions^38^. As illustrated in Fig. 4(a, b), the k–ω SST turbulence model demonstrated the closest agreement with the experimental trends across a wide range of angles of attack, with a maximum deviation of approximately 0.1% from the measured values. Based on this validation exercise, the k–ω SST model was selected for all subsequent analyses presented in this study.

### Convergence history of numerical results

A sample of convergence history of the steady RANS solution is presented in Fig. 4c. Residuals of continuity, x- and y-momentum, turbulent kinetic energy (k), and its dissipation rate (ε) were monitored. An initial rapid decay of all residuals is observed during the early iterations, followed by slower asymptotic convergence. Momentum residuals reached to (10⁻⁷ :10⁻⁸), indicating strong convergence of the velocity field, while continuity and turbulence residuals stabilized around to (10⁻⁴ :10⁻³). A transient rise in the continuity residual was encountered around iterations 300–450, this is mainly due to local flow adjustment associated with the CFJ slot coupling; the solution recovered and continued to converge. Also lift and drag monitors were tracked concurrently and exhibited stable values, confirming that the solution is converged for the purposes of the present study.

## Geometrical parameter modeling for CFJ

**Fig. 5:**
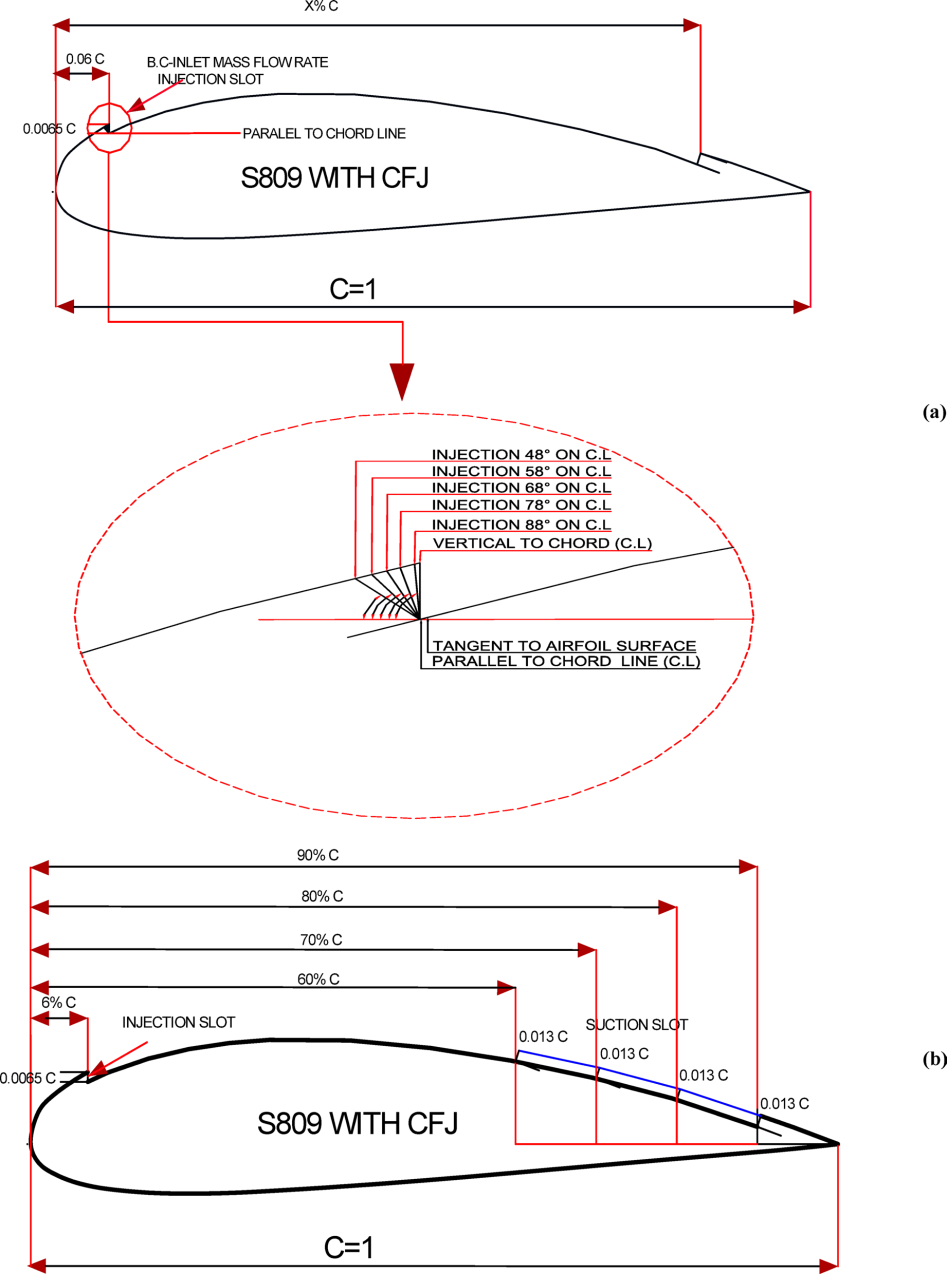
S809 airfoil with different CFJ parameters, (**a**) injection slot angles, (**b**) suction slot locations.

Enhancing the aerodynamic performance of wind turbine rotors depends significantly on several geometrical factors. Among these, the injection angle and the suction slot location play a critical role in influencing the lift (C_l_) and drag (C_d_) coefficients. This is because the injection process supplies additional momentum to the boundary layer, while the suction slot removes weak boundary regions, thereby helping to maintain flow attachment over the airfoil surface. The optimal values of injection angle and suction slot location are primarily determined by the airfoil geometry and the nominal free-stream velocity. Consequently, selecting their proper configuration can be challenging, but it is crucial for identifying the optimal airfoil design that can be effectively applied in the wind turbine industry to maximize power output. G. Zha and K. Xu^42^ conducted modeling studies on wind turbine airfoils to improve performance and identify the optimal locations and dimensions of suction and injection slots for various moment coefficients. Their results demonstrated that, with an injection slot placed at 6% of the chord and an injection pressure of approximately 1.01 atm, the lift coefficient was improved by nearly 45%. In this study, several models were developed. The first category of models was based on fixing the suction slot location at 80% of the chord and a mass flow rate equal to 2.5% of the incoming flow, while varying the injection angle at 48°, 58°, 68°, 78°, and 88°, as illustrated in Fig. 5(a). The second category of models was developed by fixing the most effective injection angle identified from the first category and maintaining the mass flow rate at 2.5%, while varying the suction slot location at 60%, 70%, 80%, and 90% of the chord, as shown in Fig. 5(b).

## Results and discussions

### Effect of injection angles

The injection angle represents a critical geometrical parameter, as it directly governs the flow direction that interacts with and energizes the weak boundary layer, thereby enhancing the effectiveness of the CFJ model. To investigate this influence, a model was developed with the suction slot located at 80% of the chord and the injection slot positioned at 6% of the chord length, while varying the injection angle at 48°, 58°, 68°, 78° and 88°, as shown in Fig. 5(a). The aerodynamic performance metrics, including drag coefficient, lift coefficient, and the lift-to-drag ratio, were evaluated. The resulting data were analyzed to determine the optimum injection angle that maximizes the lift coefficient, minimizes drag, and improves the overall aerodynamic efficiency of the airfoil. In this analysis, determining the optimum injection angle is based on two main performance indicators:

- The lift-to-drag ratio (C_l_/C_d_), which reflects the aerodynamic efficiency.
- The stall-delay capability, expressed by the increase in the angle of attack at which flow separation occurs.

**Fig. 6:**
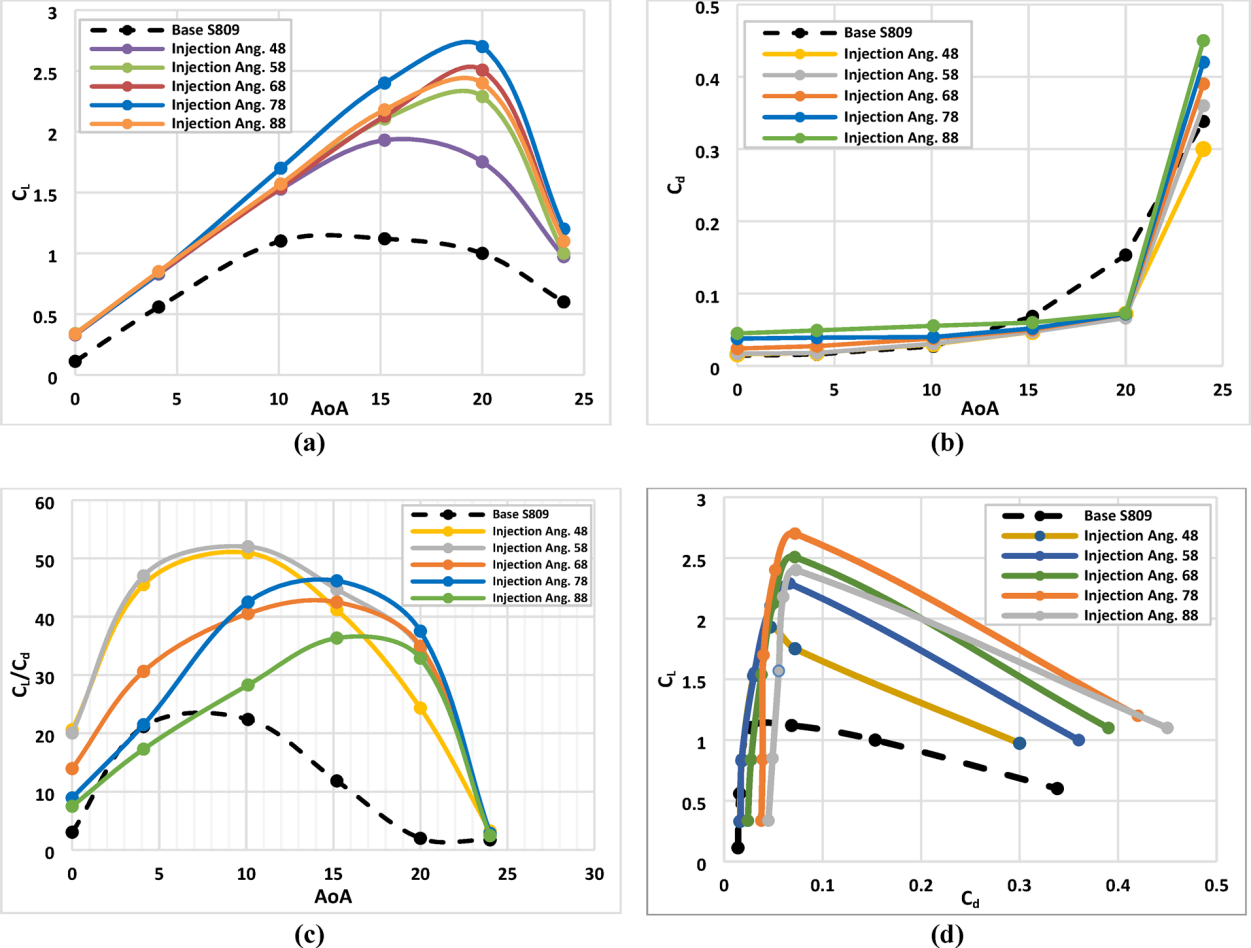
Aerodynamic characteristics of Base S-809 airfoil and Co-Flow-Jet model for different injection slot angles, (**a**) lift coefficient, (**b**) drag coefficient, (**c**) ratio of lift to drag coefficients, (**d**) lift with drag coefficient.

Figure 6(a) illustrates the variation of coefficient of lift with air flow attack angle (AoA) for various injection slot angles of 48°, 58°, 68°, 78° and 88°. From this graph we can see that the lift coefficient reaches the highest peak at injection angle of 78° and at AoA = 20° with improvement of about 170% compared with base airfoil. Figure 6(b) illustrates the drag coefficient variation with air flow angle of attack (AoA) for different injection slot angles of 48°, 58°, 68°, 78° and 88°. From this graph we can see that the drag coefficient reaches the minimum value at injection angle of 78◦ and at AoA = 20° with reduction of about 53% less than the base airfoil. Figure 6(c) illustrates the lift to drag coefficients variation with air flow angle of attack (AoA) for different injection slot angles of 48°, 58°, 68°, 78° and 88°. From this graph we can see that the C_l_/C_d_ ratio has enhanced mostly in injection angle of 78°, and at high attack angle, which means that it is the most efficient configuration of injection slot angle. Figure 6(d) illustrates the lift coefficient variation with drag coefficient for various injection slot angles of 48°, 58°, 68°, 78° and 88°. From this graph we denote that the C_l_ vs. C_d_ reaches to the max peak at injection angle of 78° which confirms that it is the most efficient configuration of injection slot angle.

From the findings when evaluating both criteria together, we observed that although the maximum C_l_/C_d_ occurs at 58°, the largest improvement in both C_l_/C_d_ and stall-delay simultaneously is achieved at 78° as illustrated in Fig. 6. At this angle, the airfoil not only maintains a significantly improved lift-to-drag ratio but also shows the greatest delay in flow separation, which is a critical performance objective in active flow control applications. Therefore, the optimum configuration will be satisfied with choosing an injection angle of about 78°.

**Fig. 7:**
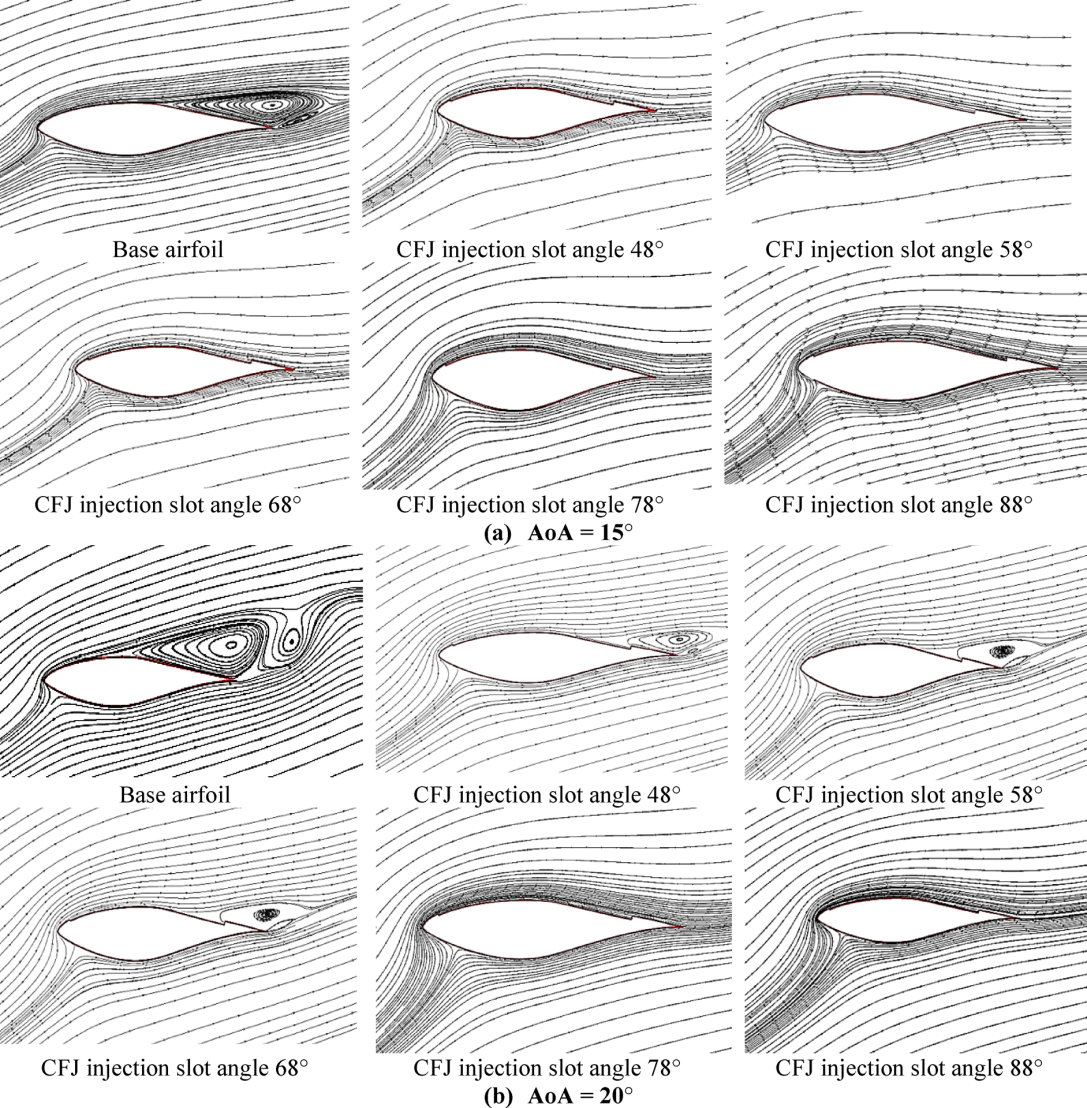
Streamline behavior of base airfoil (S809) configuration compared to CFJ configuration at two flow angles of attack and for different injection slot angles, (**a**) at AoA = 15°, (**b**) at AoA = 20°.

For understanding the effect of CFJ configurations on the airfoil characteristics of the wind turbine, the distribution of streamline flow over base airfoil and CFJ configurations are predicted at two air flow angles of attack for different injection slot angles. Figure 7 illustrates the streamline behavior comparison between the base airfoil (S809) configuration compared to the CFJ configuration at two air flow attack angles of about (AoA = 15°, and 20°) for different injection slot angles of 48°, 58°, 68°, 78° and 88°. From this graph, generally we denote that that separation layer is decreasing and demolished by increasing the injection angle up to injection angle of about 78◦ after that no clear effect can be noted also the lift coefficient starts to drop as presented in Fig. 6(a). Finally, the maximum enhancement effect of the CFJ configuration appeared at high injection angles, especially at injection angles of about 78°.

### Effect of Suction slot location

**Fig. 8:**
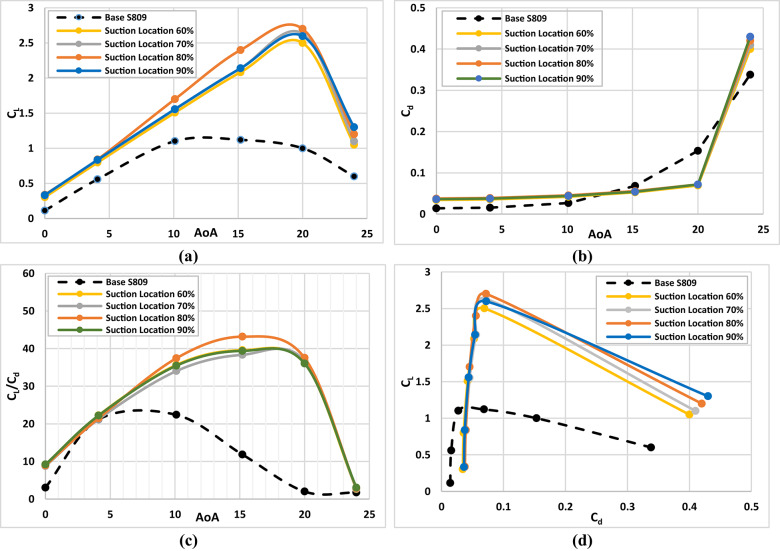
Aerodynamic characteristics of Base S-809 airfoil and Co-Flow-Jet model for different suction slot locations, (**a**) lift coefficient, (**b**) drag coefficient, (**c**) ratio of lift to drag coefficients, (**d**) lift with drag coefficient.

**Fig. 9:**
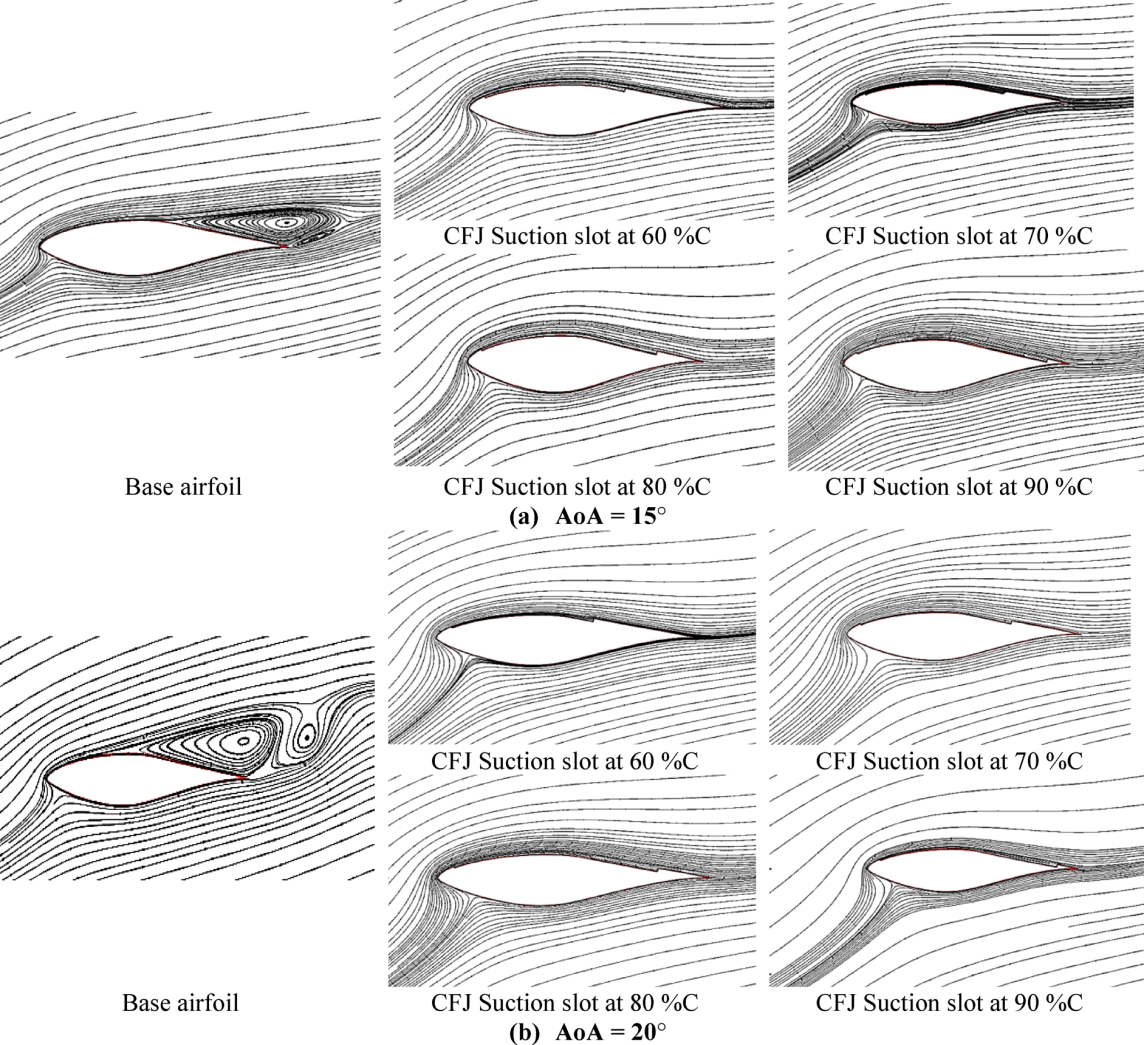
Streamline behavior of base airfoil (S809) configuration compared to CFJ configuration at two angles of attack and for different suction slot locations, (**a**) at AoA = 15°, (**b**) at AoA = 20°.

The suction slot location constitutes another critical geometrical parameter, as it determines the point at which the boundary layer is extracted, thereby influencing the stability of the CFJ airfoil configuration. Careful selection of the suction slot location is essential for generating stable and efficient CFJ airfoils. To investigate this effect, a model was developed with the injection slot positioned at 6% of the chord, an injection angle of 78°, and suction slots located at 60%, 70%, 80%, and 90% of the chord, as illustrated in Fig. 5b. The aerodynamic performance parameters, including lift coefficient, drag coefficient, and the lift-to-drag ratio, were evaluated. The results were then analyzed to identify the optimum suction slot location that maximizes lift, reduces drag, and enhances the overall aerodynamic efficiency of the airfoil. Figure 8a illustrates the variation of lift coefficient with air flow attack angle (AoA) for various suction slot locations of (60, 70, 80 and 90) %C of the chord. From this graph we can see that the lift coefficient reaches the highest peak at suction slot location of 80%C and at AoA = 20° with improvement of about 170% compared with base airfoil. Figure 8(b) shows the drag coefficients variation with air flow angle of attack (AoA) for different suction slot locations of (60, 70, 80 and 90) %C of the chord. From this graph we can see that the drag coefficient reaches the minimum value at suction slot location of 80%C and at AoA = 20° with reduction of about 53% less than the base airfoil. Figure 8c illustrates the variation of lift to drag coefficients with air flow angle of attack (AoA) for different suction slot locations of (60, 70, 80 and 90) %C of the chord. From this graph we can see that the C_l_/C_d_ ratio has enhanced mostly at suction slot location of 80%C, and at high angle of attack, which means that it is the most efficient configuration of injection slot angle. Figure 8d illustrates the lift coefficient variation with drag coefficient for various suction slot locations of (60, 70, 80 and 90) %C of the chord. From this graph we denote that the C_l_ vs. C_d_ reaches to the max peak at suction slot location of 80%C which confirms that it is the most efficient configuration of injection slot angle. From the findings it was clear that the optimum configuration will be satisfied with choosing a suction slot location of 80%C. For understanding the effect of CFJ configurations on the airfoil characteristics of the wind turbine, the distribution of streamline flow over base airfoil and CFJ configurations are predicted at two air flow angles of attack for different suction slot locations. Figure 9 illustrates the comparison of the streamline behavior between the base airfoil (S809) configuration compared to the CFJ configuration at two air flow angles of attack of about (AoA = 15°, and 20°) for different suction slot locations of (60, 70, 80 and 90) %C of the chord. From this graph, generally we denote that that separation layer is decreasing and demolished by increasing the suction slot location up to slot location of about 80%C after that no clear effect can be noted also the lift coefficient starts to drop as presented in Fig. 8a. Finally, the maximum enhancement effect of the CFJ configuration appeared at high suction slot location, especially at slot location of about 80%C.

### Effect of injected mass flow rate and turn off condition

**Fig. 10:**
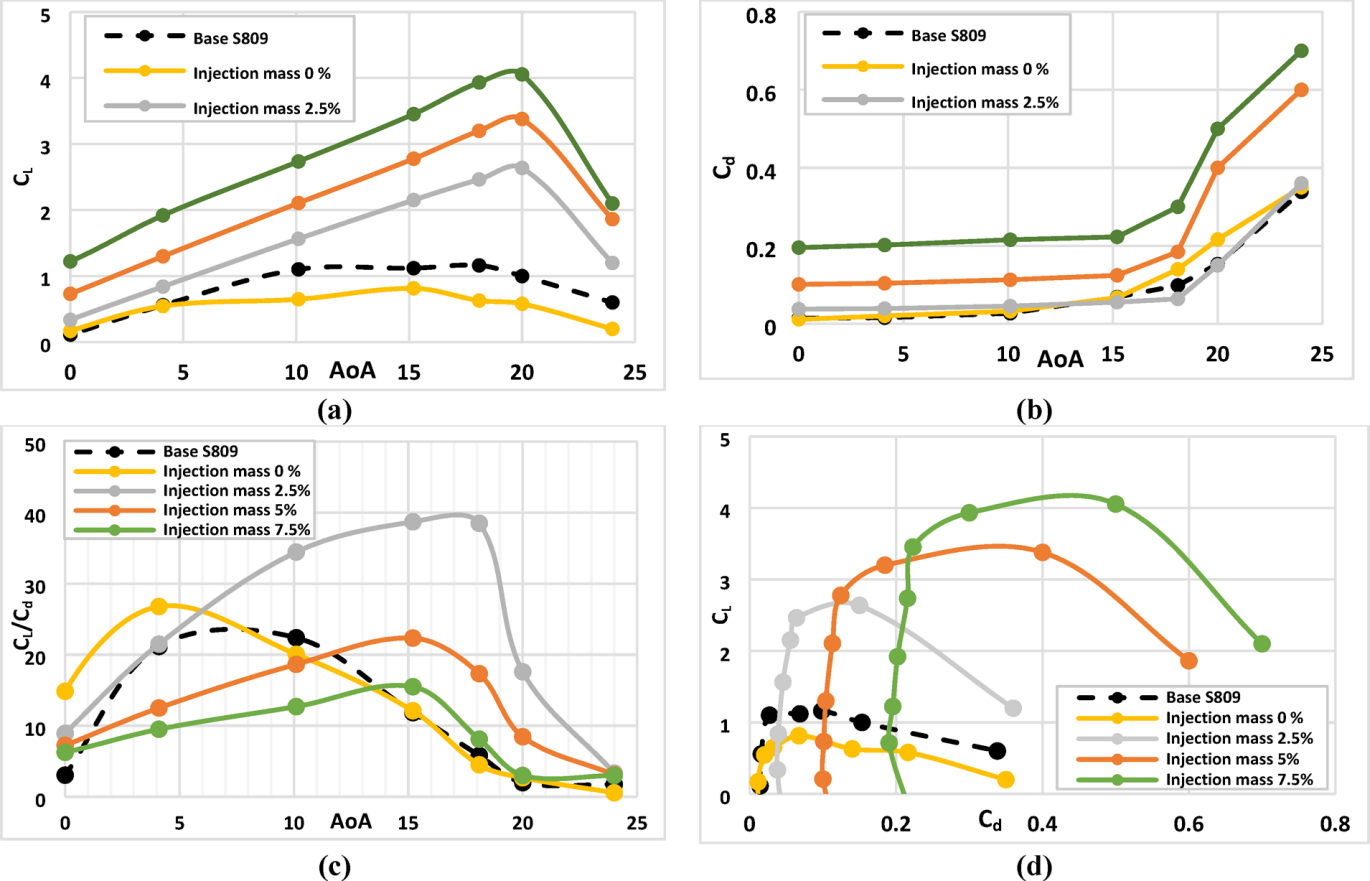
Aerodynamic characteristics of Base S-809 airfoil and Co-Flow-Jet model for different injected mass flow rate ratio, (**a**) lift coefficient, (**b**) drag coefficient, (**c**) ratio of lift to drag coefficients, (**d**) lift with drag coefficient.

**Fig. 11:**
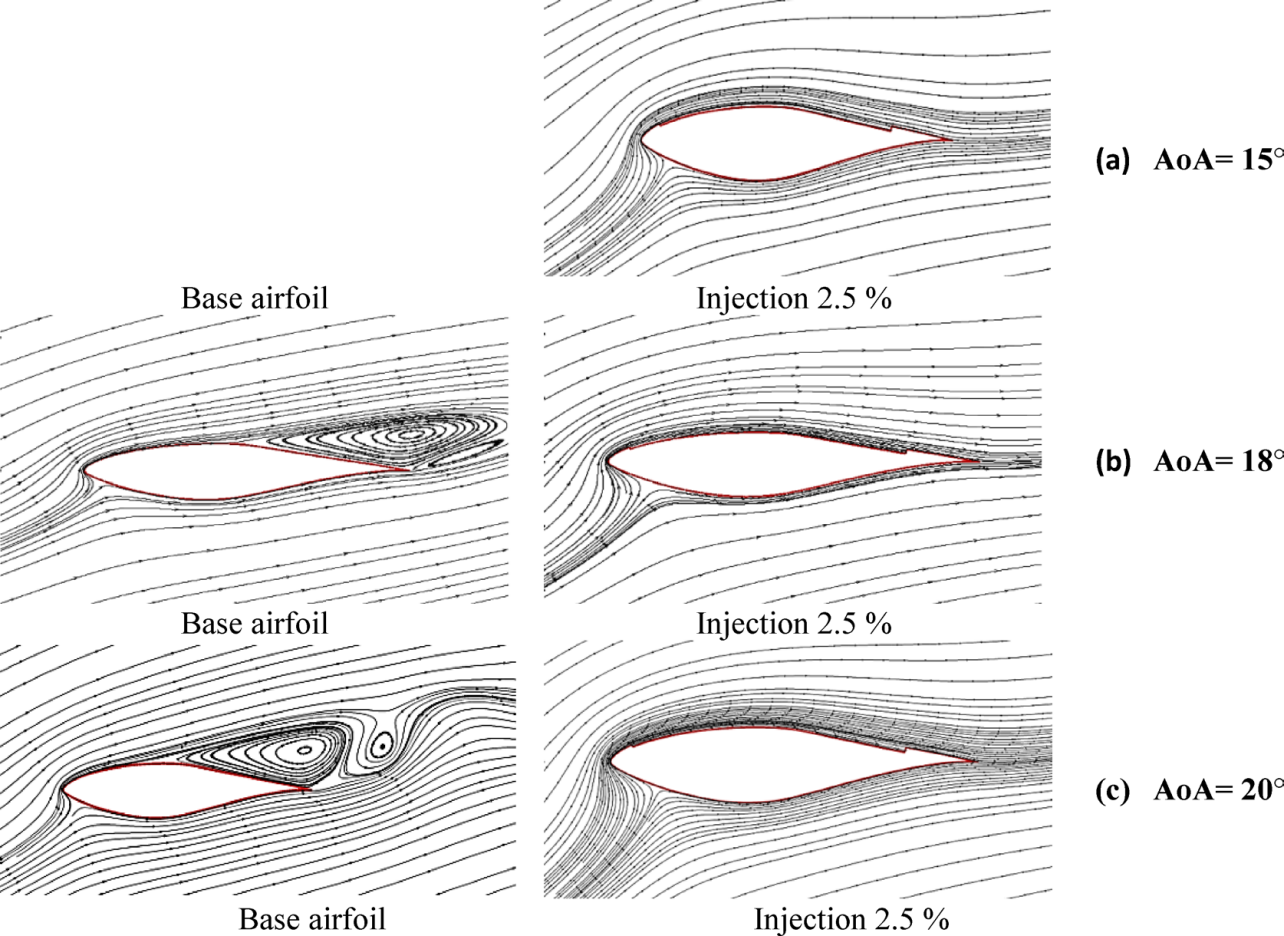
Streamline behavior of base airfoil (S809) configuration compared to CFJ configuration of injected mass flow rate ratio of 2.5% at three angles of attack, (**a**) at AoA = 15°, (**b**) at AoA = 18°, (**c**) at AoA = 20°.

In this section, the effect of the injected mass flow rate (P_3_) was examined to determine the optimal value that maximizes the aerodynamic performance of CFJ configurations. The most efficient geometrical model, identified from the previous investigation of injection angles and suction slot locations (injection angle of 78° and suction slot positioned at 80% of the chord), was selected for this analysis. The injected mass flow rate is a critical parameter, as it dictates the amount of circulated air required to stabilize the boundary layer, in addition to defining the compressor capacity necessary to operate the system under optimal conditions. Generally, the injected mass flow rate is influenced by the geometric characteristics of the airfoil model particularly the injection angle and suction slot location as well as the velocity of the incoming flow stream. For this study, the injected mass flow rate was expressed as a percentage of the upstream mass flow rate, with ratios of 2.5%, 5%, and 7.5% investigated. The incoming air flow rate (M^o^). was calculated by using Eq. (11) which equal about 14.05 kg/s. The injected mass flow rate (m^o^) was calculated using Eq. (12) according to the ratio of injected mass flow rate (P_3_ = 2.5%, 5% & 7.5%) which equal about 0.3513, 0.7026, and 1.0539 kg/s respectively. Furthermore, a 0% mass flow rate representing the compressor-off condition (turn off condition) was analyzed to evaluate the aerodynamic behavior of the CFJ configuration in the absence of active flow control. In the case of 0% mass flow rate, the slots were treated as inactive but still present on the airfoil surface to realistically represent the case in which the system stops operating while the slot geometry remains unchanged. The aerodynamic performance parameters, including the lift coefficient, drag coefficient, and lift-to-drag ratio, were assessed, and the results were analyzed to determine the optimal conditions that enhance lift, reduce drag, and improve the overall aerodynamic efficiency of the airfoil. Figure 10a illustrates the variation of lift coefficient with the air flow attack angle (AoA) for various mass flow rates ratio of 0%, 2.5%, 5%, 7.5% from the total incoming mass flow rate. From this graph we note that the coefficient of lift increases by increasing the injected rate of mass flow reaches the highest peak at AoA = 20° with improvement of about 255% compared with base airfoil. Also, in turn-off condition (0%) we noticed that the lift coefficient drop below the base airfoil. Figure 10b shows the variation of drag coefficient with air flow attack angle (AoA) for different mass flow rates ratio of 0%, 2.5%, 5%, 7.5% from the total incoming mass flow rate. On the other hand, from this graph we can see that the drag coefficient was about the same as base airfoil values in the case of injection ratio of 2.5%, after this ratio the coefficient of drag increases with increasing the injected rate of mass flow. However, the coefficient of drag at turn-off condition (0%) is more than base airfoil drag coefficient, which means that turn off condition led to drop in overall performance of CFJ airfoil this mainly due to the circulation effect of flow near the suction and injection locations. Figure 10c shows the coefficients of lift to drag variation with air flow attack angle (AoA) for different mass flow rates ratio of 0%, 2.5%, 5%, 7.5% from the total incoming mass flow rate. From this graph we can see that the C_l_/C_d_ ratio has enhanced mostly at mass flow rates ratio of 2.5%, which makes it is the most peak ratio of C_l_/C_d_ at a high angle of attack, this will support to take a decision in selecting 2.5% is considered the most efficient injection mass flow rate ratio that satisfied the optimum values of the aerodynamic characteristics of CFJ configurations. Figure 10d illustrates the lift coefficient variation with drag coefficient for various mass flow rates ratio of 0%, 2.5%, 5%, 7.5% from the total incoming mass flow rate. From this graph we denote that the C_l_ vs. C_d_ reaches to the max peak at mass flow rates ratio of 2.5% which confirms that the increasing injection rate of mass flow has little effect on the performance characteristics of CFJ airfoil configurations. For understanding the effect of the CFJ configurations on the wind turbine airfoil characteristics, the distribution of streamline flow over base airfoil and CFJ configurations are predicted at different attack angles for mass flow rates ratio of 2.5% from the total incoming rate of mass flow. Figure 11 explains the comparison of streamline behavior between the base airfoil (S809) configuration compared to the CFJ configuration at three air flow angles of attack of about (AoA = 15°, 18°, and 20°) for optimum mass flow rates ratio of 2.5% from the total incoming mass flow rate. From this graph for base S809, generally we denote that that separation layer is increasing by increasing the angles of attack, but on the other hand separation layer is decreasing and demolished by increasing angles of attack with S809 with CFJ configurations. This is mainly due to the improvement effect of CFJ configurations with (injection slot angle 78°, suction slot at 80%C) which help to delay the separation of flow at high attack angles related to the base S809 (without any modifications). Also, we can note that the streamline behavior has enhanced mostly at mass flow rates ratio of 2.5%, this will support taking a decision in selecting 2.5% is considered the most efficient injection mass flow rate ratio that enhancing the aerodynamic characteristics of CFJ configurations.

### Effect of CFJ on the pressure coefficient behaviors

**Fig. 12:**
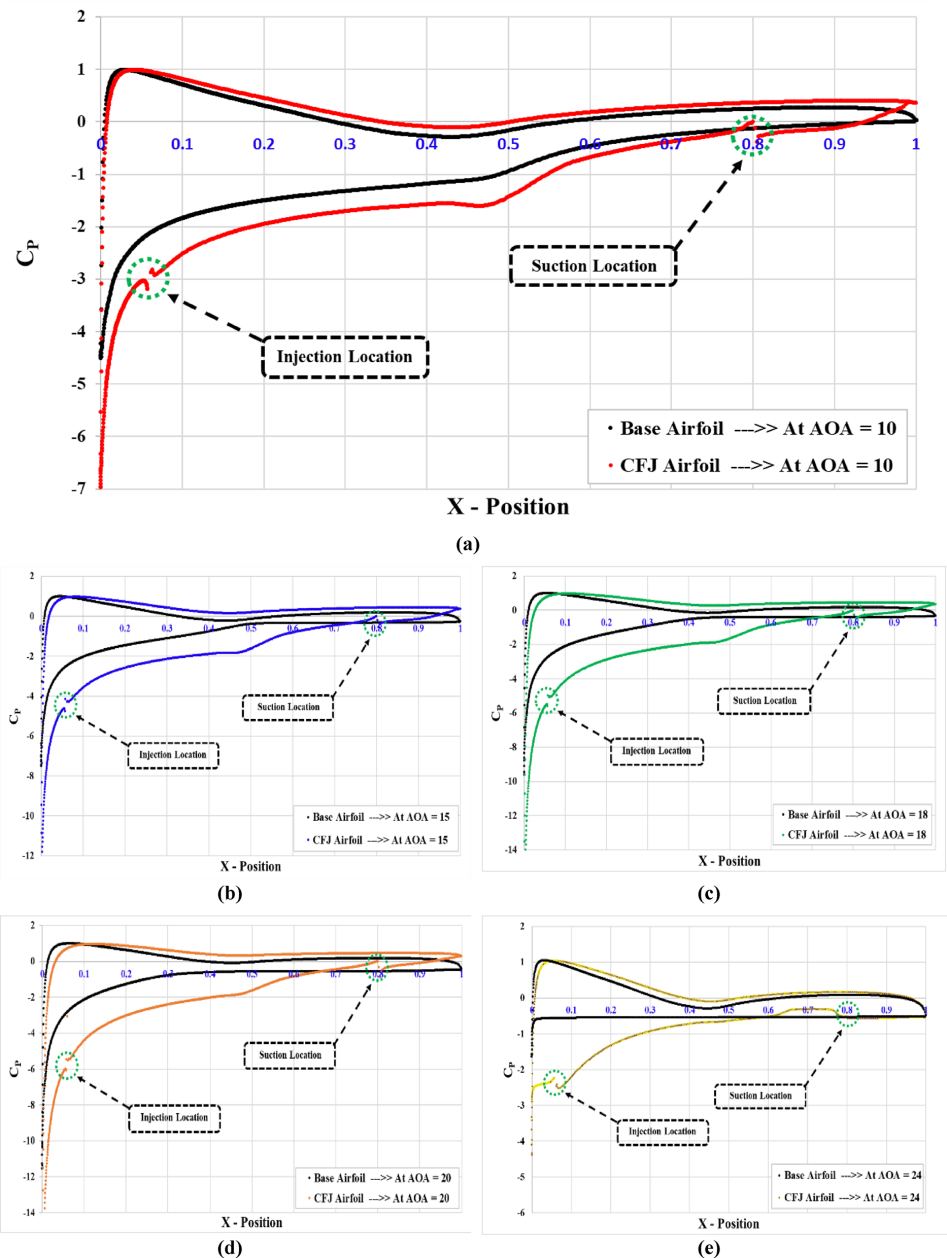
Pressure coefficient variation of base profile (S809) and optimum configurations of CFJ for different angles of attack, (**a**) at AoA = 10°, (**b**) at AoA = 15°, (**c**) at AoA = 18°, (**d**) at AoA = 20°, (**e**) at AoA = 24°.

To evaluate the influence of the CFJ technique on the aerodynamic performance of wind turbine airfoils, the pressure coefficient (C_P_) distribution was analyzed for both the baseline airfoil profile and the optimized CFJ configuration (injection slot angle of 78°, suction slot at 80% of the chord, and mass flow rate ratio of 2.5%). The variations in C_p_ were plotted along the dimensionless chord ratio (x/C) for different angles of attack (AoA = 10°, 15°, 18°, 20°, and 24°), as presented in Fig. 12. Generally, all behavior of different angles of attack shows an enhancement in pressure coefficient of airfoils with CFJ compared to the base airfoils, this is mainly because of the techniques of suction and injection of CFJ profile. In all figures we can see that there was a sharp drop in (C_P_) on the CFJ airfoil at injection area of (~ x = 0.1), this indicates enhanced suction (lower pressure), improving lift. Also, there are some disturbances at the injection point, this is mainly because of the discontinues in the airfoil surface due to the cut of injection location and injection slot angle. These disturbances have appeared also at the suction slot location (~ x = 0.8). The basic function of the suction point helps delay flow separation, maintaining better pressure recovery at the trailing edge. Generally, CFJ airfoil configurations show higher suction (more negative C_P_) than the base airfoil, resulting in higher lift and better flow control. Finally, The CFJ airfoil configurations significantly improves aerodynamic performance by increasing lift and reducing flow separation through active flow control.

## Conclusion

Delaying the stall phenomena can help to increase lift, decrease drag, enhance the overall aerodynamic characteristics of the airfoils used in wind turbines, and hence improve the overall turbine blade efficiency. The CFJ is considered as one of the most significant active flow controls techniques which is employed to migrate the generated vortices at airfoil trailing edge and hence delay stall. The CFJ was implemented on the S809 airfoil computationally by using ANSYS-Fluent package. The computational simulation domain was created, and the turbulence models were validated. The airfoils shaped without and with CFJ slots were simulated and analyzed at different suction slot’s locations and injection slot for different injected mass flow rates. The increment of drag, lift coefficients, and ratio of lift to drag between the baseline airfoil and the CFJ airfoil were compared. In general, the major findings of this study can be summarized as follows:

- The implementation of CFJ significantly improved the aerodynamic performance characteristics of the S809 airfoil used in wind turbines.
- The coefficients of lift (C_l_), coefficient of drag (C_d_), and (C_l_/C_d_) are significantly greater than those of the baseline airfoil.
- The optimum suction slot location and injection slot angle were found at about 80% chord length and 78°, respectively.
- Increasing the injected mass flow rate beyond 2.5% of the incoming upstream flow showed no additional performance improvement; the most efficient mass flow rate was 2.5%.
- Using the optimum CFJ configuration with 2.5% injection, stall was delayed from 15.2° (baseline) to 20°, representing a 31% increase in stall margin.
- The coefficient of lift (C_l_) increased by approximately 170% at AoA = 20° compared to the baseline airfoil.
- The coefficient of drag (C_d_) decreased by about 53% at AoA = 20° compared to the baseline.
- The lift-to-drag ratio (C_l_/C_d_) was substantially improved for the CFJ airfoil relative to the baseline configuration.
- Under CFJ turn-off conditions, the lift coefficient decreased by approximately 42% at AoA = 20°, drag increased, and stall occurred earlier at 16.24°, around 5° lower than the baseline.
- Finally, in industrial relevance, these improvements directly enhance wind turbine efficiency, energy capture, and operational stability under industrial working conditions.
- Moreover, the identification of optimal slot geometry and required mass-flow rates provides practical design guidelines for integrating CFJ-based active flow-control systems into commercial turbine blade development and manufacturing.

Based on the outcomes of this study and the aerodynamic enhancements achieved through the application of the CFJ technique, several avenues for further development remain open to strengthen the applicability and robustness of the proposed approach. To advance the current findings and expand their practical relevance, the following future recommendations are proposed:

- Further investigation of CFJ implementation on other airfoil profiles used in horizontal-axis wind turbines is recommended to generalize the observed aerodynamic improvements.
- Experimental validation of the optimum CFJ configurations identified in this study is suggested to confirm the computational predictions and assess real-world performance under varying wind conditions.
- Optimization of CFJ system design, including compressor energy consumption and slot geometry, is recommended to achieve higher overall turbine efficiency while minimizing energy costs.

## Data Availability

Data is provided within the manuscript file.
